# Exploring Nonantibiotic Adjuncts: Synergistic Effects of Esmolol and Carvedilol With Tigecycline

**DOI:** 10.1155/cjid/1402388

**Published:** 2026-04-10

**Authors:** Mehmet Erinmez, İpek Koçer, Yasemin Zer

**Affiliations:** ^1^ Faculty of Medicine, Department of Medical Microbiology, Gaziantep University, Gaziantep, Türkiye, gantep.edu.tr; ^2^ Department of Medical Microbiology, SANKO University Faculty of Medicine, Gaziantep, Türkiye

**Keywords:** antimicrobial resistance, carvedilol, checkerboard, drug repurposing, esmolol, synergy, tigecycline

## Abstract

**Background:**

Antimicrobial resistance poses a major global health threat, and carbapenem‐resistant Gram‐negative bacteria such as *Klebsiella pneumoniae* and *Acinetobacter baumannii* have been classified as critical priority pathogens. Although tigecycline provides coverage against multidrug resistant organisms, its use is limited by pharmacokinetic constraints and the emergence of resistance. This study investigates the in vitro synergistic potential of tigecycline combined with two β‐blockers, carvedilol and esmolol, against clinical isolates of *K. pneumoniae*, *Proteus mirabilis*, and *A. baumannii*.

**Methods:**

Synergy was assessed using the disc diffusion, broth microdilution, and checkerboard methods. A total of 36 clinical isolates were tested. Esmolol and carvedilol were supplemented with tigecycline discs, and inhibition zone diameters were compared. Minimum inhibitory concentrations (MICs) and fractional inhibitory concentration indices (FICi) were calculated according to standard protocols.

**Results:**

Both β‐blockers significantly enhanced tigecycline’s antibacterial activity across all isolates, as evidenced by increased inhibition zones and synergistic FICi values (≤ 0.5). Neither carvedilol nor esmolol exhibited intrinsic antimicrobial activity at therapeutic concentrations, suggesting their synergistic role is not based on direct bactericidal effects. These findings support the hypothesis that β‐blockers modulate bacterial susceptibility, possibly by interfering with quorum sensing or membrane dynamics.

**Conclusion:**

Carvedilol and esmolol significantly potentiate tigecycline’s efficacy against MDR Gram‐negative pathogens in vitro. These results support repurposing β‐blockers as nonantibiotic adjuvants in antimicrobial therapy. Further molecular studies and in vivo validation are warranted to explore their potential in clinical applications and to better understand the mechanisms underlying the observed synergy.

## 1. Introduction

Antimicrobial resistance (AMR) represents a major global health challenge, and, if urgent measures are not implemented, it is estimated to result in up to 10 million deaths each year by 2050 [1]. The World Health Organization (WHO) has listed *Acinetobacter baumannii* and carbapenem‐resistant *Enterobacteriaceae* among its top‐priority pathogens, emphasizing the need for alternative therapies [2]. The global rise of carbapenem‐resistant Gram‐negative bacteria, especially those producing carbapenemases, underscores the urgency of developing adjunctive treatments [3, 4]. Tigecycline, a glycylcycline antibiotic, is effective against multidrug‐resistant (MDR) pathogens and is used in complex polymicrobial and nosocomial infections [5]. However, resistance and pharmacokinetic limitations constrain its monotherapy use [6].

Drug repurposing, re‐evaluating approved drugs for new indications, offers a faster and cost‐effective alternative to traditional antibiotic development [7, 8]. Recent efforts focus on combining nonantibiotic agents with antimicrobials to improve efficacy, especially against MDR bacteria [9]. β‐adrenergic receptor antagonists (β‐blockers), widely used for cardiovascular conditions, exhibit antioxidant, calcium channel‐blocking, and cardiodepressant effects [10]. Their established safety and accessibility make them strong candidates for repositioning in infectious disease treatment.

β‐blockers reduce myocardial oxygen consumption, blood pressure, and heart rate by blocking β‐adrenoceptors, effects that are especially beneficial in critical care settings [10, 11]. They may also have antimicrobial roles. Propranolol, for instance, disrupts quorum sensing (QS), attenuating bacterial virulence without applying direct selective pressure [12]. Carvedilol exhibits activity against a broad spectrum of bacteria, including both Gram‐positive and Gram‐negative species and may interfere with microbial metabolism [13, 14]. Esmolol has demonstrated anti‐inflammatory and antiapoptotic effects in septic models [15]. This study investigates the in vitro synergy between tigecycline and two β‐blockers, carvedilol and esmolol, against key bacterial pathogens. Findings may support the repurposing of these agents as antimicrobial adjuvants, offering a novel strategy to combat AMR.

## 2. Materials and Methods

### 2.1. Bacterial Isolates

Guided by the results of a priori power analysis, a total of 36 clinical isolates were included in the study. Twelve isolates each of *Klebsiella pneumoniae*, *Proteus mirabilis*, and *A. baumannii* were randomly selected from blood culture samples obtained from patients treated at a tertiary care university hospital in southeast Türkiye between May 2023 and February 2025. To ensure randomization, resistance profiles, sample type, and hospital ward were not used as selection criteria. All eligible isolates were assigned unique identification numbers, and random selection was performed using a computer‐generated random number sequence generated in Microsoft Excel. Only one nonduplicate isolate per patient was included. *Escherichia coli* ATCC 25922 *and Pseudomonas aeruginosa* ATCC 27853 were employed as reference strains for quality control [16]. Bacterial identification was conducted with the Vitek2 platform (bioMérieux, France).

### 2.2. Disc Diffusion Method

The disc diffusion technique was used to investigate possible synergy between tigecycline and the β‐blockers carvedilol and esmolol. *K. pneumoniae*, *P. mirabilis*, and *A. baumannii* isolates preserved in skim milk medium at −20°C allowed to thaw at ambient temperature and subsequently inoculated onto 5% sheep blood agar (BD, Germany). Bacterial suspensions matching a 0.5 McFarland standard were prepared using colonies obtained from agar plates incubated for 24 h, following the direct colony suspension method. Inoculation was performed by evenly spreading the suspension on Mueller Hinton Agar (MHA; Oxoid, Boston, USA) using a sterile swab. Carvedilol and esmolol were dissolved in dimethyl sulfoxide (DMSO), filtered through a 0.22 μm syringe filter, and stored at −80°C until use. For each isolate, three tigecycline discs (Bioanalyse, Ankara, Turkey) were placed on the agar plates:

1. Control tigecycline disc
2. Tigecycline disc supplemented with 10 μL of 10 mg/mL carvedilol (Arlec; Ali Raif İlaç, Türkiye)
3. Tigecycline disc supplemented with 10 μL of 10 mg/mL esmolol (Esmobloc; Polifarma, Türkiye).

Plates were incubated at 35 ± 2°C for 24 h, and the diameters of the inhibition zones were subsequently measured. Interpretation of zone diameters followed the EUCAST guidelines [16].

### 2.3. Broth Microdilution Method

The antibacterial activity of tigecycline, carvedilol, and esmolol was assessed using the broth microdilution method according to ISO 20776‐1:2006. All experiments were conducted in cation‐adjusted Mueller Hinton Broth (MHB). Carvedilol and esmolol were tested across a concentration range of 0.25–2048 mg/L. A wide concentration range was selected to allow detection of potential concentration‐dependent interactions. Tigecycline (Carbosynth, Campton, UK) was tested across a range of 0.25–64 mg/L. Broth microdilution assays were performed in 96‐well plates. A bacterial suspension prepared in CAMHB was added to each well to achieve a final concentration of 5 × 10^5^ CFU/mL. The plates were incubated at 35 ± 2°C for 24 h. The minimum inhibitory concentration (MIC) was defined as the lowest concentration of antimicrobial agent that visibly inhibited bacterial growth.

### 2.4. Checkerboard Method

The checkerboard method was used to evaluate the interaction between tigecycline and each β‐blocker by calculating the fractional inhibitory concentration index (FICi). Serial twofold dilutions of tigecycline were prepared along the columns, and serial twofold dilutions of carvedilol or esmolol were prepared along the rows of 96‐well plates. Each well was inoculated with a bacterial suspension in cation‐adjusted MHB to achieve a final inoculum of 5 × 10^5^ CFU/mL. Plates were incubated at 35 ± 2°C for 24 h. FICi was calculated using the formula: FICi = (MIC of drug A in combination/MIC of drug A alone) + (MIC of drug B in combination/MIC of drug B alone).

Interpretation of FICi values was as follows [17]:

1. FICi ≤ 0.5: synergy
2. 0.5 < FICi < 1: additive
3. 1 ≤ FICi ≤ 4: indifferent
4. FICi > 4: antagonism

When the MIC for a compound exceeded the highest concentration tested, the next twofold higher concentration was used for calculation (e.g., if MIC > 16 μg/mL, 32 μg/mL was used) [18].

### 2.5. Statistical Analyses

The sample size was guided by an a priori power analysis using G∗Power (version 3.1), assuming a two‐sided alpha level of 0.05 and a statistical power of 80%. Based on effect sizes reported in previous in vitro antimicrobial interaction studies, 12 isolates per bacterial species were considered adequate for the planned comparisons. Statistical analyses were conducted to compare inhibition zone diameters. Normality was assessed using the Kolmogorov–Smirnov and Shapiro–Wilk tests, while Levene’s test evaluated the equal variances assumption. Based on data distribution, comparisons were performed using either the Student’s *t*‐test for normally distributed data or the Wilcoxon rank sum test for data that were not normally distributed. Descriptive statistics are presented as mean ± standard deviation (M ± SD) for variables following a normal distribution and as median with first (Q1) and third quartiles (Q3) for variables with nonnormal distributions. All analyses were carried out using IBM SPSS Statistics for Windows version 24.0 (IBM Corp., USA). A *p* value of less than 0.05 was considered statistically significant.

## 3. Results

Synergistic interactions were observed between tigecycline and both esmolol and carvedilol in the *K. pneumoniae*, *P. mirabilis*, and *A. baumannii* isolates. Supplementation with esmolol and carvedilol led to a notable increase in inhibition zone diameters with statistical significance of tigecycline (Tables 1 and 2).

**TABLE 1:** Inhibition zone diameters of tigecycline with and without esmolol.

Isolate	Inhibition zone diameters (mm)				Mean increase of inhibition zone diameters (95% CI) control vs. +esmolol	*p* value	*t* value
	Control		+Esmolol				
	Mean	SD	Mean	SD			
*K. pneumoniae* (*n*:12)	21.5	± 2.07	24	± 1.56	2.5 [−4.02, −0.98]	**0.0026** ^t^	−3.39
*A. baumannii* (*n*:12)	19.5	± 0.90	23.2	± 2.18	3.6 [−5.07, −2.27]	**< 0.001** ^t^	−5.39
*P. mirabilis* (*n*:12)	18.2	± 1.76	22.7	± 1.96	4.5 [−5.84, −3.16]	**< 0.001** ^t^	−6.21

*Note:* The *p* values in bold are statistically significant.

Abbreviations: 95% CI, 95% confidence interval; SD, standard deviation.

^t^ *t*‐test.

**TABLE 2:** Inhibition zone diameters of tigecycline with and without carvedilol.

Isolate	Inhibition zone diameters (mm)				Mean increase of inhibition zone diameters (95% CI) control vs. +Carvedilol	*p* value	*t* or *Z*
	Control (*n* = 11)		+Carvedilol				
	Mean/median	SD/Q1; Q3	Mean/median	SD/Q1; Q3			
*K. pneumoniae* (*n*:12)	20.5	20.0; 22.7	23.5	22.0; 28.0	3.0 [0.34, 5.66]	**0.028** ^W^	−2.20
*A. baumannii* (*n*:12)	19.5	± 0.90	23.33	± 1.72	3.8 [‐4.71, −2.79]	**< 0.001** ^t^	−7.14
*P. mirabilis* (*n*:12)	18.2	± 1.86	24.25	± 2.73	6.0 [‐7.83, −4.17]	**< 0.001** ^t^	−6.37

*Note:* The *p* values in bold are statistically significant. Q1: 1st quartile; Q3: 3rd quartile.

Abbreviations: 95% CI, 95% confidence interval; SD, standard deviation.

^t^ *t*‐test.

^W^Wilcoxon rank sum test.

However, no intrinsic bacteriostatic or bactericidal effects were observed for esmolol and carvedilol at their respective human therapeutic concentrations (esmolol: 0.3 mg/kg; carvedilol: 100 mg/day) against the strains screened in this research [19, 20]. Outcomes of antimicrobial susceptibility evaluations obtained via the broth microdilution method were consistent with those from the disc diffusion test. Checkerboard assays revealed a synergistic interaction between tigecycline and both esmolol and carvedilol across all *K. pneumoniae*, *P. mirabilis*, and *A. baumannii* isolates evaluated in the study (Table 3).

**TABLE 3:** Broth microdilution and checkerboard synergy test results.

Isolates	Tigecycline MIC (mg/L)	Esmolol MIC (mg/L)	Tig + Esm FICi and int.	Carvedilol MIC (mg/L)	Tig + Car FICi and int.
KP1	1	> 2048	0.5: Synergy	> 2048	0.5: Synergy
KP2	1	> 2048	0.5: Synergy	> 2048	0.5: Synergy
KP3	1	> 2048	0.5: Synergy	> 2048	0.5: Synergy
KP4	1	> 2048	0.5: Synergy	> 2048	0.5: Synergy
AB1	2	> 2048	0.25: Synergy	> 2048	0.25: Synergy
AB2	4	> 2048	0.125: Synergy	> 2048	0.25: Synergy
AB3	4	> 2048	0.25: Synergy	> 2048	0.125: Synergy
AB4	1	> 2048	0.5: Synergy	> 2048	0.5: Synergy
PM1	4	> 2048	0.125: Synergy	> 2048	0.125: Synergy
PM2	16	> 2048	0.031: Synergy	> 2048	0.031: Synergy
PM3	8	> 2048	0.062: Synergy	> 2048	0.062: Synergy
PM4	8	> 2048	0.062: Synergy	> 2048	0.25: Synergy

*Note:* Int: interpretation; Tig: tigecycline; Esm: esmolol; Car: carvedilol.

Abbreviations: AB, *A. baumannii*; FICi, fractional inhibitory concentration index; KP, *K. pneumoniae*; PM, *P. mirabilis*.

## 4. Discussion

The increasing prevalence of MDR bacterial infections poses a significant challenge to current antimicrobial therapies, necessitating innovative strategies beyond traditional antibiotics. This study demonstrates that the β‐blockers esmolol and carvedilol potentiate the antibacterial activity of tigecycline against clinically relevant Gram‐negative pathogens, including *K. pneumoniae*, *P. mirabilis*, and *A. baumannii*. The observed synergistic effect, characterized by a notable enhancement in antimicrobial activity, was evidenced by increased inhibition zone diameters and confirmed through checkerboard assays. These findings align with emerging evidence supporting the repurposing of nonantibiotic drugs as adjuvants in antimicrobial therapy [7]. Notably, neither esmolol nor carvedilol exhibited intrinsic bacteriostatic or bactericidal effects at therapeutic human doses, indicating that their primary role is not direct bacterial killing but rather modulation of bacterial susceptibility. This finding is consistent with previous reports suggesting that β‐blockers may interfere with bacterial virulence and communication systems without exerting selective pressure that typically drives resistance [21]. As a limitation, there is no standardized guideline for defining the upper concentration limits of nonantibiotic agents in checkerboard synergy assays; therefore, concentration ranges are commonly determined based on exploratory study objectives, compound solubility, and prior screening data [22]. Also, the in vitro concentrations tested do not correspond to plasma concentrations attainable in clinical settings. Importantly, the synergistic concentrations identified in checkerboard assays (≥ 2048 mg/L) are several orders of magnitude higher than the reported peak plasma concentrations (*C*_max_) of carvedilol (≈0.02–0.1 mg/L) and esmolol (≈0.8–1.5 mg/L) [23, 24]. This highlights a substantial translational gap between in vitro findings and clinically achievable exposures.

At the molecular level, bacterial pathogens sense host adrenergic hormones such as norepinephrine to regulate virulence via QS and other signaling pathways. β‐blockers are hypothesized to disrupt this bacterial “espionage” by antagonizing adrenergic receptor‐like sensors, thereby diminishing virulence factor expression and pathogenicity [25, 26]. These findings support this model, as the synergistic enhancement of tigecycline activity may reflect impaired bacterial adaptation and survival mechanisms [21]. Supporting this hypothesis, carvedilol has been shown to enhance ciprofloxacin efficacy through disruption of calcium homeostasis, morphological alterations, and inhibition of bacterial aggregation in *Staphylococcus aureus* [27]. Similarly, propranolol, another β‐blocker, reduces QS‐controlled virulence traits such as biofilm formation and protease production in *P. aeruginosa* and *Serratia marcescens*, further emphasizing the potential of β‐blockers to modulate bacterial behavior beyond growth inhibition [12]. Furthermore, biophysical studies reveal that β‐blockers like propranolol and acebutolol interact electrostatically with bacterial membranes, fluidizing lipid bilayers and disrupting membrane integrity [28]. These membrane‐targeted effects could synergize with tigecycline’s ribosomal inhibition by increasing drug uptake or impairing bacterial defense mechanisms.

In conclusion, this study underscores the potential of esmolol and carvedilol to enhance in vitro antimicrobial activity of tigecycline against Gram‐negative bacteria. While the underlying mechanisms were not directly investigated, these results contribute to a growing body of evidence positioning β‐blockers as promising nonantibiotic adjuncts that could potentiate existing antibiotics and help mitigate resistance development. Future research should focus on elucidating the precise molecular interactions between β‐blockers and bacterial targets, optimizing dosing regimens, and validating efficacy in in vivo infection models.

## Funding

No funding was received for this study.

## Ethics Statement

Ethical approval for the study was granted by the Gaziantep University Clinical Research Ethics Committee (Approval No. 2025/218, dated July 2, 2025).

## Conflicts of Interest

The authors declare no conflicts of interest.

## Data Availability

The data that support the findings of this study are available from the corresponding author upon reasonable request.
